# Nonsurgical Management of Submental Fullness for Double Chin Reduction: A Systematic Review

**DOI:** 10.1111/jocd.70925

**Published:** 2026-06-22

**Authors:** Khatere Zahedi, Mehdi Gheisari

**Affiliations:** ^1^ Skin Research Center Shahid Beheshti University of Medical Sciences Tehran Iran; ^2^ Department of Dermatology, Loghman Hakim Hospital Shahid Beheshti University of Medical Sciences Tehran Iran

**Keywords:** aesthetic dermatology, ATX‐101, deoxycholic acid, double chin, submental fat

## Abstract

**Background:**

Submental fullness (SMF), commonly known as a “double chin,” is a prevalent aesthetic concern that can affect facial harmony and self‐perception. A range of surgical and nonsurgical treatment options are available for SMF reduction, including deoxycholic acid injections, energy‐based devices, and liposuction. However, a comprehensive synthesis of current evidence on the efficacy and safety of these interventions is lacking.

**Aims:**

This study aimed to systematically evaluate the effectiveness, safety, and clinical outcomes of various nonsurgical interventions used to reduce double chin.

**Patients/Methods:**

This systematic review was conducted in accordance with PRISMA guidelines. A comprehensive search of PubMed, Scopus, Web of Science, Embase, and Google Scholar was performed for studies published from January 2010 to March 2025. Out of 1805 initial records, 30 studies met the inclusion criteria. These included randomized controlled trials, prospective cohorts, and observational studies.

**Results:**

The most frequently evaluated treatment was injectable deoxycholic acid (ATX‐101), followed by cryolipolysis, radiofrequency, high‐intensity focused ultrasound, and surgical liposuction. Injectable lipolysis with ATX‐101 demonstrated consistent efficacy, with ≥ 1‐grade improvement in SMF in the majority of patients and satisfaction rates between 67% and 86%. Energy‐based devices showed promising results with minimal downtime and high tolerability. Surgical interventions were effective but less commonly preferred. Emerging agents such as DWJ211, Tapencarium, and ascorbyl‐palmitate‐based solutions also showed potential in early‐phase trials. Most adverse events were mild, transient, and localized.

**Conclusions:**

Injectable deoxycholic acid is currently the most studied and effective nonsurgical treatment for SMF reduction. Energy‐based modalities offer viable alternatives with favorable safety profiles. Surgical methods remain effective but are less desirable due to invasiveness.

## 1 Introduction

The submental area is a key factor of overall facial balance and harmony. Submental fullness (SMF), commonly referred to as a double chin, is a prevalent aesthetic concern that can significantly impact facial harmony and self‐perception. It has been stated that reduction of SMF can not only enhance facial aesthetics but also contribute to improved self‐esteem and overall psychological well‐being [1, 2, 3]. The 2019 Consumer Survey conducted by the American Society for Dermatologic Surgery revealed that 73% of the 3645 participants expressed concern about excess fat in the chin and neck area [4].

SMF reduction can be achieved through a variety of treatment strategies, encompassing surgical procedures or nonsurgical/minimally invasive methods [3, 5]. For individuals with considerable SMF, skin laxity, or a desire for quicker and more noticeable improvements, surgical options such as liposuction, neck lift, or submentoplasty (chin tuck) may be recommended [6, 7]. Although traditional liposuction remains an effective approach, several noninvasive alternatives such as deoxycholic acid injections (Kybella, Allergan Aesthetics, an AbbVie company, USA), cryolipolysis (CoolSculpting), laser lipolysis (SculpSure), ultrasound‐based therapy (Ultherapy), and radiofrequency treatments have emerged, providing patients with effective options that involve minimal downtime and reduced procedural risks [3, 8, 9, 10, 11].

Deoxycholic acid (DCA), commercially known as ATX‐101, is an injectable medication approved by the US Food and Drug Administration (FDA) in 2015 for the nonsurgical reduction of submental fat (SMF). It is a minimally invasive, effective, and well‐tolerated treatment for adults with mild to moderate SMF. Clinical experience indicates that most individuals opting for DCA treatment present with less severe submental fullness, reflecting a growing preference for nonsurgical aesthetic options. However, DCA is not ideal for patients with significant skin laxity or complex anatomical features, though it remains a valuable alternative to surgical procedures for suitable candidates [11, 12, 13, 14, 15].

Among energy‐based modalities, several technologies including diode lasers, radiofrequency (RF), and cryolipolysis have been investigated for their ability to safely and effectively reduce SMF. Diode laser treatments work by inducing targeted lipolysis through controlled heat, while RF devices stimulate collagen remodeling and fat reduction by delivering thermal energy to deeper tissue layers. Cryolipolysis, on the other hand, reduces localized fat deposits by controlled cooling and subsequent adipocyte apoptosis. More recently, a novel system combining high‐intensity focused electromagnetic stimulation (HIFES) with synchronized RF has demonstrated promising results, with significant decreases in fat thickness observed in both preclinical and early clinical studies [3, 5, 16].

The present systematic review aims to offer a more comprehensive and up‐to‐date synthesis of all currently available nonsurgical/minimally invasive methods for the reduction or removal of SMF, encompassing both surgical and nonsurgical approaches.

## 2 Methods

This systematic review was prepared according to the Preferred Reporting Items for Systematic and Meta‐Analysis (PRISMA) guidelines [17]. The protocol for this review has not been registered with any organization. All the steps including searching, selection of included papers, and quality assessment of articles were performed by two authors independently, and any discrepancies were resolved through discussion or consultation with a third reviewer.

### 2.1 Search Strategy

A comprehensive literature search was conducted across multiple electronic databases, including PubMed, Scopus, Web of Science, Embase, and Google Scholar. The search included studies published in English between January 1, 2010, and March 30, 2025, with the final search performed on March 30, 2025. In addition to electronic searches, reference lists of relevant studies were manually screened to identify any additional eligible publications.

The search strategy incorporated both controlled vocabulary (e.g., MeSH and Emtree terms) and free‐text keywords. Boolean operators (AND, OR) were applied to refine and combine search concepts, and truncation (e.g., “prolong”*) was used to capture word variants. The detailed search strategy for each database is presented in Table A.1.

### 2.2 Eligibility Criteria

Observational studies and clinical trials were included. Exclusion criteria included case reports, animal studies, review articles, duplicate publications, studies with low methodological quality, or those lacking sufficient data for analysis.

### 2.3 Study Selection

All retrieved references were imported into EndNote (v20) for duplicate removal. Two researchers independently screened the titles and abstracts of identified studies. Titles and abstracts were screened independently by two reviewers according to predefined inclusion and exclusion criteria. Any disagreements were resolved through discussion, and when consensus could not be reached, a third reviewer was consulted to provide a final decision. The PRISMA 2020 flow diagram was used to document the study selection process.

### 2.4 Data Extraction

From the eligible studies, key data were extracted, including the author(s), year of publication, study design, sample size, type of intervention (surgical, nonsurgical, or combined), follow‐up duration, treatment outcomes, reported adverse events, and overall conclusions. All data were systematically recorded using a standardized data extraction form, and a descriptive analysis was conducted to summarize and interpret the findings.

### 2.5 Risk of Bias Assessment

The quality of observational cohort studies was assessed using the nine‐point Newcastle‐Ottawa Scale (NOS), which evaluates studies across three key domains: selection, comparability, and outcome [18]. Studies scoring 7 or more stars were classified as high quality. For clinical trials, the Jadad Scale (Oxford quality scoring system) was employed—a straightforward tool that rates studies based on randomization, blinding, and the handling of withdrawals or dropouts, with scores ranging from 0 to 5 [19].

### 2.6 Statistical Analysis

Data from the included studies were extracted and synthesized using a random‐effects meta‐analysis model to account for between‐study variability. Pooled estimates were reported as proportions or effect sizes with corresponding 95% confidence intervals (CIs). Heterogeneity across studies was assessed using the *I* ^2^ statistic, with values greater than 75% indicating substantial heterogeneity. If a high degree of heterogeneity was observed (*I* ^2^ > 90%), publication bias was not formally assessed using funnel plots or Egger's regression test, as the significant heterogeneity and limited number of comparable studies made such plots unreliable and potentially misleading. Instead, a qualitative evaluation of publication bias was conducted by examining study characteristics, sample size distribution, and methodological consistency.

All statistical analyses were performed using Comprehensive Meta‐Analysis (CMA) software, and results were presented as forest plots to visualize the pooled effect estimates and confidence intervals.

## 3 Results

### 3.1 Study Overview

**FIGURE 1:**
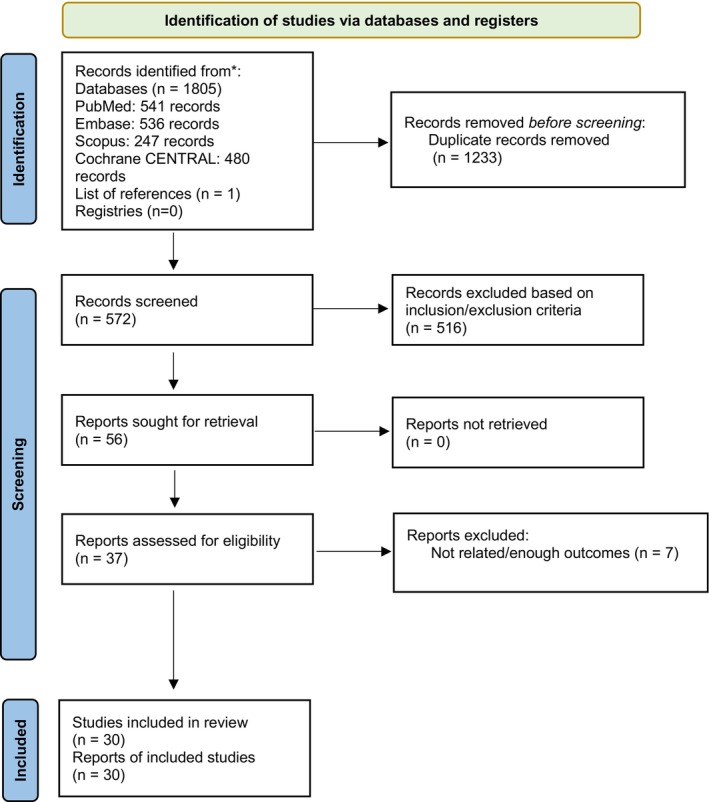
Flowchart of study selection.

Figure 1 illustrates the process of search and study selection procedure. This systematic review included 30 studies (randomized controlled trials, prospective cohorts, and observational studies) evaluating nonsurgical and minimally invasive methods for submental fullness (SMF) reduction. The interventions most frequently assessed were injectable deoxycholic acid (DCA/ATX‐101; 17 studies), cryolipolysis (11 studies), radiofrequency (3 studies), high‐intensity focused ultrasound (HIFU; 1 study), diode laser (1 study), novel injectables (e.g., DWJ211, RZL‐012; 2 studies), a combined Sync RF+ modality (1 study), and surgical liposuction (2 studies). Study sample sizes ranged from 6 to 676 participants; the CONTOUR registry reported *N* = 676 (ATX‐101: 570; energy devices: 77; surgical: 23). A summary of the key data extracted from the included studies is provided in the Table 1.

**TABLE 1:** Summary of the key data extracted from the included studies.

Author, year	Country	Applied method	Study design	Participants no	Treatment/follow‐up duration	Outcomes
Ascher et al. 2014 [20]	USA	DCA (ATX‐101 1–2 mg/cm^2^) injection	Clinical trial (Phase 3) double‐blind, placebo‐controlled	*n* = 360	Up to four treatments ~28 days apart, with a 12‐week follow‐up.	More than 58% achieved at least a one‐point improvement in fat reduction, and over 64% reported satisfaction with their facial and chin appearance. Secondary outcomes also showed improved self.
Azuelos et al. 2019 [21]	France	High‐intensity focused ultrasound (HIFU)	Prospective clinical trial (5 blinded evaluators)	*n* = 20	NA	Four out of five evaluators rated improvements in double chin appearance and skin laxity as significant. No major complications occurred, and pain levels ranging from 2.05 to 5.6 (low–medium)
Beer et al. 2019 [22]	USA	DCA ATX‐101 (2 mg/cm^2^) injection	Open‐label clinical trial	*n* = 165	Up to four treatments ~28 days apart, with a 12‐week follow‐up.	Most participants achieved at least a one‐grade SMF improvement (86.8% clinician‐assessed; 83.8% self‐assessed), with results maintained at 12 months (90.4% and 80.7%, respectively). Overall, 84.9% were satisfied with their appearance, and 93% showed stable or improved skin laxity.
Bernstein and Bloom 2017 [23]	USA	Cryolipolysis	Prospective, nonrandomized interventional cohort study	*n* = 14	NA	13 participants (93%) were satisfied Adverse effects of the procedure were typically mild and included numbness and tingling, which resolved without intervention by the final 12‐week follow‐up.
Gusmão et al. 2020 [24]	Brazil	Cryolipolysis	Single‐arm, nonrandomized trial	*n* = 20	NA	91% of patients were satisfied.
Dover et al. 2016 [25]	USA	DCA injection	Double‐blind, parallel‐group, exploratory Phase 3b study	*n* = 83	Single session and days 28 (±5 days) and 84 (±7 days) follow‐ups	Results from this study support the safety of ATX‐101 for SMF reduction, and demonstrate that pain and bruising associated with ATX‐101 treatment can be mitigated by a series of simple measures.
Dover et al.2018 [26]	USA	DCA injection	Double‐blind, parallel‐group, exploratory Phase 3b study	*n* = 83	Single session and days 28 (±5 days) and 84 (±7 days) follow‐ups	By day 84, efficacy rate based on clinician assessment significantly increased from 14.1% to 47.0%, and efficacy rate based on patient satisfaction rose slightly from 67.7% to 71.2% Most side effects after were related to the injection site and included swelling/edema (100%), pain (100%), induration (99%), bruising/hemorrhage (96%), and discomfort (68%)
Ge et al. 2025 [27]	USA	DCA + low‐dose triamcinolone acetonide (TAC) and lidocaine injection	Double‐blind, randomized clinical trial	*n* = 15	One session/Days 1, 30, 60, 90, and 180 follow‐ups	The addition of low‐dose TAC and lidocaine to DCA injections is a safe and effective method to enhance patient comfort and treatment outcomes without inducing steroid‐related side effects.
Gibson et al. 2024 [28]	USA	DCA (ATX‐101: KYBELLA) injection	Retrospective cross‐sectional study	*n* = 21	Mixed/NA	Reported adverse events included excessive swelling (24%), marginal mandibular nerve weakness and unsatisfactory aesthetic outcomes (both 19%), numbness (14%), and less common issues such as dysphagia, infection, and skin necrosis (each 5%–14%). Notably, two patients with underlying systemic conditions required hospitalization due to skin necrosis.
Glogau et al. 2019 [29]	USA	DCA (ATX‐101) injection	Clinical trial (Phase 3) double‐blind, placebo‐controlled trial	*n* = 101	≤ 6 treatments/follow‐up at 12‐week	ATX‐101 is an effective and generally well‐tolerated nonsurgical treatment for SMF reduction, with the 0.5% and 1.0% concentrations offering the best balance between efficacy and tolerability.
Goldberg 2025 [30]	USA	Noninvasive simultaneous delivery of HIFES and synchronized radiofrequency+ (Sync RF+)	Pilot study	*n* = 6	Single 20‐min treatment/24 h and 7 days follow‐ups	Caspase‐7 levels increased by 511% at 24‐h posttreatment, and 101% at 7 days (*p* < 0.0001), while the Bcl‐2 levels decreased by 89% at 24 h and 24% at 7 days posttreatment (*p* < 0.0001). The control group had no statistically significant relative changes in the activity of caspase‐7.
Goodman et al. 2021 [31]	Australia/UK/USA	DCA ATX‐101 (0.5%, 1.0%, or 2.0%) injection	Randomized, Placebo‐Controlled trial	*n* = 84	≤ 4 treatments every 28 days/With a 16‐week follow‐up.	ATX‐101 proved effective and well‐tolerated for SMF reduction, with 0.5%–1.0% concentrations showing optimal efficacy and safety.
Humphrey et al. 2021 [32]	USA	DCA (ATX‐101) injection	Double‐blind, parallel‐Phase 3 studies (REFINE‐1 and REFINE‐2)	*n* = 224	12 weeks after their last session	Most subjects showed at least a one‐grade improvement in SMF, with 86.4% confirmed by clinicians after 1 year, 90.6% after 2 years Significant reductions from baseline in psychological impact scores were sustained through 3 years (*p* < 0.001). Adverse events were mostly mild, transient, and localized.
Jones et al. 2016 [33]	USA	DCA (ATX‐101) injection	Randomized, double‐blind, placebo‐controlled, Phase 3 trial	*n* = 256	12 weeks after their last session	ATX‐101 significantly improved psychological well‐being and treatment satisfaction (*p* < 0.001), with clinician‐assessed fat reduction in 55% of patients after two treatments and 75% after four. Adverse events were mostly mild, transient, and localized, including a 4.3% rate of temporary marginal mandibular nerve paresis.
Kim et al. 2023 [34]	South Korea	Micro‐insulated needle radiofrequency (RF)	Prospective, single‐center, single‐arm, observational study	*n* = 24	1 and 2 months after last session	The average volume of submental fat was significantly decreased after 2 months (20.44 ± 5.53 cc to 16.41 ± 4.58 cc, *p* < 0.001). Patient satisfaction was high. Transient and mild local skin reactions without long‐term sequelae were observed in 4 patients.
Leal et al. 2017 [35]	Mexico	Cryolipolysis	Single‐arm, nonrandomized trial	*n* = 15	2 treatments with an interval of 10 weeks/follow‐up at 12 weeks after second treatment	Statistically significant reduction in SMF and patients' high satisfaction.
Kilmer et al. 2016 [36]	USA	Cryolipolysis	Single‐arm, nonrandomized trial	*n* = 60	6 weeks after the initial treatment.	Ultrasound showed a 2.0 mm average fat reduction. Most patients were satisfied (83%), with 80% recommending the treatment and 77% noting visible improvement. The procedure was well‐tolerated (76% comfort), with no serious adverse events reported.
Suh et al. 2017 [37]	South Korea	Cryolipolysis	Single‐arm, nonrandomized trial	*n* = 10	Follow‐up after 8 weeks	Statistically significant reduction in SMF and patients expressed high satisfaction.
Paik et al. 2022 [38]	South Korea	Injection of DWJ211 (a newly developed lipolytic injectable)	Randomized, double‐blind, multi‐center, placebo‐ Phase 2 clinical trial	*n* = 136	Every 4 weeks, up to Week 12.	The 1% DWJ211 dose was beneficial for SMF reduction and had a tolerable safety profile.
Palm et al. 2019 [5]	Australia/UK/Italy/Singapore	ATX‐101 injection/energy‐based device/surgical liposuction Laser liposuction	Prospective observational study (CONTOUR)	N total = 676 ATX‐101 = 570 Energy‐based device = 77 Surgical liposuction = 23 Laser liposuction = 5 Other treatments = 9	Patients were followed until treatment completion, discontinuation, or 1 year elapsed from enrollment without treatment	The majority of patients were at least partially satisfied with results, regardless of the chosen treatment. Physicians most frequently cited a preference for a noninvasive/minimally invasive procedure as the reason for choosing either ATX‐101 or energy‐based devices.
Park et al. 2016 [39]	South Korea	Radiofrequency (RF) technology	Prospective, single‐center, single‐arm, observational study	*n* = 21	1 and 6 months after the last treatment (0, 2, and 7 months).	At 2‐ and 7‐months post‐treatment, 82.3% and 52.9% of patients, respectively, demonstrated more than mild improvement. The average pain score indicated moderate discomfort. No significant adverse effects were reported.
Rauso 2018 [40]	Italy	DCA (ATX‐101) mixed with lidocaine 2%	Prospective, single‐center, single‐arm, observational study	*n* = 12	Follow‐up at 2 months after last session	All patients showed visible skin tightening by the second month. Minor bruising resolved within 10 days, and one case of temporary dysesthesia resolved spontaneously. No nerve injury, hair loss, or other complications occurred, and no patients required pain medication.
Rzany et al. 2014 [41]	Germany/UK	DCA (ATX‐101) injection	Clinical trial (Phase 3) double‐blind, placebo‐controlled	*n* = 363	Up to four treatment sessions ~28 days apart, with a 12‐week follow‐up.	Patient satisfaction with facial and chin appearance (SSRS) was significantly higher in the ATX‐101 groups (53.3% and 66.1%) versus 28.7% in the placebo group (*p* < 0.001). Caliper measurements confirmed a statistically significant reduction in submental fat (*p* < 0.001), and importantly, there was no observed worsening in skin laxity.
Scarano et al. 2023 [42]	Italy	Injection of lipolytic solution containing sodium salt of ascorbic acid at 0.24% and a surfactant agent at 0.020% ascorbyl‐palmitate (SAP)	Prospective, single‐center, single‐arm, observational study	*n* = 10	Total of 4 sessions, with biweekly procedures	Improvement in submental appearance was achieved in 90% (9/10) of the patients. One patient did not see any improvement in submental appearance after two section treatments.
Shome et al. 2019 [43]	India	Generic DCA injection	Prospective, single‐center, single‐arm, observational study	*n* = 50	12‐week follow‐up period/sessions were spaced approximately 2 months apart.	Reduction in SMF as confirmed by caliper measurements was statistically significant.
Shridharani 2019 [44]	USA	DCA (ATX‐101) injection	Prospective, single‐center, single‐arm, observational study	*n* = 100	Follow‐up at 1 and 5 to 7 weeks after last session	Deoxycholic acid injections were generally well tolerated, and ≥ 2 treatment sessions were required to achieve the desired aesthetic goal in a private practice setting.
Shridharani et al. 2023 [45]	USA	Injection of Tapencarium (novel injectable synthetic molecule with cytolytic properties)	3‐armed, randomized, double‐blind, placebo‐controlled phase 2b study	*n* = 151	Follow‐up at 84 days	The relative percentage reduction in MRI‐measured SMF volume (Day 84 vs. screening) was significantly greater in the high‐dose RZL‐012 group vs. the low‐dose RZL‐012 or the placebo group (*p* < 0.0001). Local injection site reactions were the most common adverse events (AEs)
Valizadeh, et al. 2016 [46]	Iran	980‐nm diode laser with the power of 6–8 W/traditional liposuction	Randomized prospective controlled clinical trial	*n* = 36 (18 each group)	Follow‐up at 2 weeks and 2 months after the procedures.	Significant reduction of fat thickness in each group compared with the baseline (*p*‐value < 0.001). At the 2 weeks and 2 months follow‐up visit, fat thickness reduction was significantly higher in the lipolysis group (*p*‐value < 0.05).
Yuan et al. 2022 [47]	USA	DCA (ATX‐101) injection	Prospective, single‐center, single‐arm, observational study	*n* = 15	4 treatment sessions/follow‐up 12 weeks after last session	On the 6‐point Subject Self‐Rating Scale, patients showed an average improvement of +3.0 points, and 86% reported being satisfied with the treatment.
Zarbafian et al. 2020 [48]	Iran	DCA (ATX‐101) injection	Prospective, single‐center, single‐arm, observational study	*n* = 81	Mixed/NA	The average Subject Goal Aesthetic Improvement Scale scores were 2.7 after the first treatment and 2.25 after the second, showing a significant improvement (*p* = 0.01). About 67% of patients were “somewhat” or “very” satisfied with the results. Side effects were temporary and only occurred in the treated area.

Abbreviations: CR‐SMFRS, Clinician‐Reported Submental Fat Rating Scale; DCA, deoxycholic acid; HIFU, high‐intensity focused ultrasound; NA, not available; PR‐SMFRS, Patient‐Reported Submental Fat Rating Scale.

### 3.2 Meta‐Analysis

Based on the extracted data from the included studies, nonsurgical interventions for SMF demonstrated favorable outcomes in terms of both effectiveness and safety. The pooled analysis of clinical trials revealed a mean responder rate of approximately 70%–75%, indicating substantial improvement in submental contour and patient satisfaction across modalities. Deoxycholic acid (DCA; ATX‐101) showed the most consistent efficacy, with significant reductions in SMF thickness and improved aesthetic appearance after multiple treatment sessions.

#### 3.2.1 DCA Injection

**FIGURE 2:**
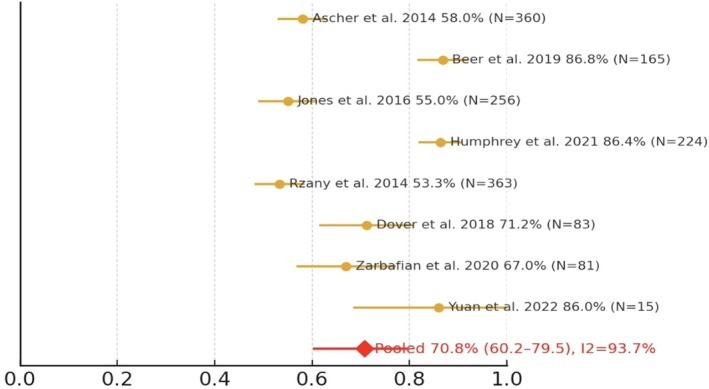
Forest plot of improvement incidence for DCA injection. Pooled proportion = 70.8% (95% CI: 60.2–79.5); *I* ^2^ = 93.7%.

Table 2 summarizes the study‐level responder proportions for deoxycholic acid (DCA; ATX‐101) across eight clinical studies, comprising a total of 1547 participants, with individual sample sizes ranging from 15 to 363. As shown in Figure 2, the pooled analysis demonstrated a responder proportion of 70.8% (95% CI: 60.2–79.5), indicating that approximately seven out of 10 patients achieved a clinically meaningful improvement in SMF reduction following DCA treatment. The *I* ^2^ value of 93.7% reflects substantial heterogeneity among studies, likely attributable to differences in study design, dosing protocols, follow‐up duration, and outcome assessment methods.

**TABLE 2:** Extracted study‐level proportions for DCA injection.

Study	Sample size (no)	Events	Satisfaction (%)
Ascher et al. 2014	360	209	58.0%
Beer et al. 2019	165	143	86.8%
Jones et al. 2016	256	141	55.0%
Humphrey et al. 2021	224	194	86.4%
Rzany et al. 2014	363	193	53.3%
Dover et al. 2018	83	59	71.2%
Zarbafian et al. 2020	81	54	67.0%
Yuan et al. 2022	15	13	86.0%

### 3.3 Risk of Bias (RoB) Assessment

Tables 3 and 4 shows the risk of bias (RoB) assessment for the included studies, using RoB 2 for randomized trials (Table 3) and ROBINS‐I for nonrandomized studies (Table 4).

**TABLE 3:** Risk of bias (RoB) assessment for randomized controlled trials (Cochrane RoB 2 tool).

Author, Year	Study type	Bias due to confounding	Bias in selection of participants	Bias in classification of interventions	Bias due to deviations from intended interventions	Bias due to missing data	Bias in measurement of outcomes	Bias in selection of the reported result	Overall risk
Ascher et al. 2014	RCT (Phase 3, DB, PC)	🟩	🟩	🟩	🟩	🟩	🟩	🟩	🟩
Dover et al. 2016	RCT (DB)	🟩	🟩	🟩	🟩	🟩	🟩	🟩	🟩
Dover et al. 2018	RCT (DB)	🟩	🟩	🟩	🟩	🟩	🟩	🟩	🟩
Ge et al. 2025	RCT (DB)	🟩	🟩	🟩	🟩	🟩	🟩	🟩	🟩
Glogau et al. 2019	RCT (DB, PC)	🟩	🟩	🟩	🟩	🟩	🟩	🟩	🟩
Goodman et al. 2021	RCT (DB, PC)	🟩	🟩	🟩	🟩	🟩	🟩	🟩	🟩
Humphrey et al. 2021	RCT (DB)	🟩	🟩	🟩	🟩	🟩	🟩	🟩	🟩
Jones et al. 2016	RCT (DB, PC)	🟩	🟩	🟩	🟩	🟩	🟩	🟩	🟩
Paik et al. 2022	RCT (DB, PC)	🟩	🟩	🟩	🟩	🟩	🟩	🟩	🟩
Rzany et al. 2014	RCT (DB, PC)	🟩	🟩	🟩	🟩	🟩	🟩	🟩	🟩
Shridharani et al. 2023	RCT (DB, PC)	🟩	🟩	🟩	🟩	🟩	🟩	🟩	🟩
Valizadeh et al. 2016	RCT	🟩	🟩	🟩	🟩	🟩	🟩	🟩	🟩

**TABLE 4:** Risk of bias (RoB) assessment for nonrandomized and observational studies (ROBINS‐I tool).

Author, Year	Study type	Bias due to confounding	Bias in selection of participants	Bias in classification of interventions	Bias due to deviations from intended interventions	Bias due to missing data	Bias in measurement of outcomes	Bias in selection of the reported result	Overall risk
Azuelos et al. 2019	Prospective trial (non‐RCT)	🟨	🟩	🟩	🟩	🟩	🟨	🟩	🟨
Beer et al. 2019	Open‐label clinical trial	🟨	🟩	🟩	🟨	🟩	🟨	🟩	🟨
Bernstein and Bloom 2017	Nonrandomized cohort	🟨	🟩	🟩	🟩	🟩	🟨	🟩	🟨
Gusmão et al. 2020	Nonrandomized trial	🟨	🟩	🟩	🟩	🟩	🟨	🟩	🟨
Kim et al. 2023	Prospective non‐RCT	🟨	🟩	🟩	🟩	🟩	🟨	🟩	🟨
Leal et al. 2017	Nonrandomized trial	🟨	🟩	🟩	🟩	🟩	🟨	🟩	🟨
Kilmer et al. 2016	Nonrandomized trial	🟨	🟩	🟩	🟩	🟩	🟨	🟩	🟨
Suh et al. 2017	Nonrandomized trial	🟨	🟩	🟩	🟩	🟩	🟨	🟩	🟨
Goldberg 2025	Pilot (non‐RCT)	🟥	🟨	🟩	🟩	🟩	🟥 (biomarker only)	🟩	🟥
Gibson et al. 2024	Retrospective	🟥	🟨	🟩	🟩	🟨	🟨	🟩	🟥
Palm et al. 2019	Observational	🟥	🟨	🟩	🟩	🟩	🟨	🟩	🟥
Park et al. 2016	Observational	🟨	🟩	🟩	🟩	🟩	🟨	🟩	🟨
Rauso 2018	Observational	🟨	🟩	🟩	🟩	🟩	🟨	🟩	🟨
Scarano et al. 2023	Observational	🟨	🟩	🟩	🟩	🟩	🟨	🟩	🟨
Shome et al. 2019	Observational	🟨	🟩	🟩	🟩	🟩	🟨	🟩	🟨
Shridharani 2019	Observational	🟨	🟩	🟩	🟩	🟩	🟨	🟩	🟨
Yuan et al. 2022	Observational	🟨	🟩	🟩	🟩	🟩	🟨	🟩	🟨
Zarbafian et al. 2020	Observational	🟨	🟩	🟩	🟩	🟩	🟨	🟩	🟨

Of the 30 included studies, randomized trials showed low risk of bias across all domains, indicating strong methodological rigor. In contrast, most nonrandomized and observational studies were assessed as having moderate risk of bias, mainly due to potential confounders and the subjective nature of outcome assessments. A smaller number of studies, particularly retrospective analyses and some experimental trials, were assessed as having serious risk of bias, often related to selection bias, lack of control groups, or limited outcome measures.

Overall, the evidence base is strengthened by consistent low risk ratings across RCTs, but findings from nonrandomized designs should be interpreted with caution due to concerns about moderate to serious bias.

## 4 Discussion

To the best of our knowledge this is the first comprehensive systematic review collecting and analyzing data on various nonsurgical/minimally invasive modalities for removing/reducing SMF. Based on the extracted data from the included studies, nonsurgical interventions for SMF demonstrated favorable outcomes in terms of both effectiveness and safety. Among nonsurgical treatments, deoxycholic acid (DCA; ATX‐101) is the most widely studied agent, demonstrating consistent efficacy in improving submental contour with an approximate 70% response rate. However, due to heterogeneity in study design and the limited number of comparative trials, these findings should not be interpreted as evidence of superior efficacy compared with other available methods. Cryolipolysis and radiofrequency‐based treatments also yielded notable reductions in submental volume, though typically requiring repeated sessions to achieve optimal results.

Reported adverse events were predominantly mild and transient, including localized swelling, numbness, erythema, and tenderness, with serious complications being rare. Across studies, incidence rates of adverse effects remained below 10%, supporting the favorable safety profile of these approaches. Despite some heterogeneity among study protocols, follow‐up durations, and outcome measures, the overall evidence supports the efficacy, tolerability, and patient satisfaction associated with nonsurgical treatments for double chin reduction. Although surgical approaches are less well‐studied, they remain the preferred choice for patients seeking faster, more noticeable results. In contrast, nonsurgical treatments are gaining popularity among those who prefer less invasive methods with shorter recovery times.

Data on injectable lipolysis using DCA/ATX101 showed high efficacy in reducing SMF. Patient satisfaction rates generally between 67% and 86%. Efficacy of DCA injection was demonstrated in multiple phase III randomized controlled trials (e.g., REFINE‐1 and REFINE‐2) in the United States and Canada of 1022 adults with moderate or severe SMF [32]. Administered of DCA usually is performed at 2 mg/cm^2^, up to 50 injections per session, spaced 1 cm apart with up to six sessions can be performed at ≥ 1‐month intervals [49]. DCA/ATX101 causes adipocyte lysis when injected into subcutaneous fat, leading to the breakdown and clearance of fat cells via inflammatory and macrophage‐mediated processes. It also promotes fibrosis and potential neocollagenesis, contributing to improved skin contouring. It has rapid systemic absorption, with plasma levels peaking within 18 min and returning to baseline within 24 h [50]. A few studies reported rare but serious events like skin necrosis and nerve paresis. ATX‐101 was associated with a higher incidence of expected treatment‐related adverse events such as pain, erythema, swelling, edema, numbness, and fibrosis, particularly at the 2 mg/cm^2^ dose. Most side effects were transient, localized, and mild to moderate in severity, with resolution typically occurring within 28 days consistent with the inflammatory and adipocytolytic mechanism of the drug [20, 22, 31, 33, 43, 47]. Due to the potential for bleeding complications, caution is advised when using ATX‐101 in individuals with bleeding disorders or those on anticoagulant or antiplatelet therapy. It is generally recommended to delay treatment until platelet counts or coagulation parameters normalize.

Data from 11 on energy‐based techniques indicated that these modalities are generally effective for reducing SMF. Cryolipolysis showed fat layer reduction (~2 mm via ultrasound) and high satisfaction rates up to 93% [23]. RF and HIFU also demonstrated improvements in both SMF and skin tightening [21, 34]. Data from included studies demonstrated that MFU is an effective noninvasive treatment for mild‐to‐moderate facial skin laxity. Objective outcomes, including brow and submental lift measurements, showed measurable skin tightening effects—such as a mean submental area reduction of 26–45 mm^2^ and brow lift improvements ranging from 0.47 to 1.7 mm. These improvements were often sustained for up to one year post‐treatment [51, 52]. Pain was reported across studies, with mean scores ranging from 2.5 to 6.1, particularly higher in submandibular and periorbital regions. However, most adverse effects such as erythema, swelling, or mild bruising were transient and self‐limiting. More serious side effects (e.g., dysesthesia, burns, striations) were rare, occurring in only 2% of patients [5, 46, 52].

Data from the CONTOUR study by Palm et al., which evaluated a range of SMF reduction techniques including both surgical and nonsurgical methods, highlighted several key factors influencing patient decision‐making. Cost emerged as the most significant consideration when choosing to undergo treatment. The selection of a specific treatment modality was primarily guided by the severity of SMF and the patient's preference for nonsurgical versus surgical approaches. Additionally, the growing availability of noninvasive or minimally invasive options has made SMF reduction a popular entry‐point procedure for individuals new to facial aesthetic treatments [5].

### 4.1 Limitations

This systematic review has several limitations. First, considerable heterogeneity was observed in the included studies in terms of design, sample size, follow‐up duration, and outcome measures, which may limit the comparability of results and contribute to variability in effect estimates. Second, most of the included studies were open‐label, nonrandomized, or industry‐sponsored, which poses a risk of bias and potential overestimation of treatment effectiveness. Third, the lack of head‐to‐head comparative trials prevents definitive conclusions about the relative effectiveness of different nonsurgical methods.

## 5 Conclusion

Nonsurgical treatments for submental fullness, such as deoxycholic acid injections, cryolipolysis, and energy‐based devices, provide effective and minimally invasive options for reducing submental fat. Deoxycholic acid shows consistent efficacy with a pooled improvement rate of about 70%. While deoxycholic acid remains the most extensively studied intervention, evidence on emerging modalities remains limited and warrants further investigation through high‐quality randomized controlled trials to confirm their safety, durability, and relative effectiveness.

## Funding

The authors have nothing to report.

## Ethics Statement

The authors have nothing to report.

## Conflicts of Interest

The authors declare no conflicts of interest.

## Data Availability

The data that support the findings of this study are available from the corresponding author upon reasonable request.

## Appendix A

**TABLE A.1:** Detailed database search strategies.

Database	Search strategy/query
PubMed	((“Submental Fat”[Mesh] OR “submental fat”[tiab] OR “double chin”[tiab] OR “submental fullness”[tiab] OR “chin fat”[tiab]) AND (“Non‐surgical”[tiab] OR “nonsurgical”[tiab] OR “non invasive”[tiab] OR “noninvasive”[tiab] OR “minimally invasive”[tiab] OR “non‐surgical management”[tiab] OR “non‐surgical treatment”[tiab]) AND (“Treatment”[tiab] OR “therapy”[tiab] OR “management”[tiab] OR “reduction”[tiab] OR “removal”[tiab] OR “lipolysis”[tiab] OR “deoxycholic acid”[tiab] OR “ATX‐101”[tiab] OR “cryolipolysis”[tiab] OR “radiofrequency”[tiab] OR “ultrasound”[tiab] OR “laser”[tiab] OR “Kybella”[tiab])) NOT (“Surgery”[tiab] OR “surgical”[tiab] OR “liposuction”[tiab])
Scopus	TITLE‐ABS‐KEY(“submental fat” OR “double chin” OR “submental fullness” OR “chin fat”) AND TITLE‐ABS‐KEY(“non‐surgical” OR “nonsurgical” OR “non invasive” OR “noninvasive” OR “minimally invasive”) AND TITLE‐ABS‐KEY(“treatment” OR “therapy” OR “management” OR “reduction” OR “removal” OR “lipolysis” OR “deoxycholic acid” OR “ATX‐101” OR “cryolipolysis” OR “radiofrequency” OR “ultrasound” OR “laser” OR “Kybella”) AND (PUBYEAR > 2009 AND PUBYEAR < 2026)
Web of Science	TS = (“submental fat” OR “double chin” OR “submental fullness” OR “chin fat”) AND TS = (“non‐surgical” OR “nonsurgical” OR “non invasive” OR “noninvasive” OR “minimally invasive”) AND TS = (“treatment” OR “therapy” OR “management” OR “reduction” OR “removal” OR “lipolysis” OR “deoxycholic acid” OR “ATX‐101” OR “cryolipolysis” OR “radiofrequency” OR “ultrasound” OR “laser” OR “Kybella”) NOT TS = (“surgery” OR “surgical” OR “liposuction”)
Embase	(“submental fat”/exp. OR “submental fat”:ti,ab OR “double chin”:ti,ab OR “submental fullness”:ti,ab OR “chin fat”:ti,ab) AND (“non surgical”:ti,ab OR “nonsurgical”:ti,ab OR “non invasive”:ti,ab OR “noninvasive”:ti,ab OR “minimally invasive”:ti,ab) AND (“treatment”/exp. OR “therapy”/exp. OR “management”/exp. OR “reduction”/exp. OR “lipolysis”/exp. OR “deoxycholic acid”/exp. OR “atx 101”:ti,ab OR “cryolipolysis”/exp. OR “radiofrequency”/exp. OR “ultrasound”/exp. OR “laser”/exp. OR “kybella”:ti,ab) NOT (“surgery”/exp. OR “surgical”:ti,ab OR “liposuction”/exp)
